# Evaluation of Compensation Strategies for Gait Impairment in Patients With Parkinson Disease

**DOI:** 10.1212/WNL.0000000000201159

**Published:** 2022-11-15

**Authors:** Anouk Tosserams, Noël Keijsers, Willanka Kapelle, Roy P.C. Kessels, Vivian Weerdesteyn, Bastiaan R. Bloem, Jorik Nonnekes

**Affiliations:** From the Departments of Rehabilitation (A.T., N.K., W.K., V.W., J.N.), and Neurology (A.T., W.K., B.R.B.), Center of Expertise for Parkinson & Movement Disorders, Donders Institute for Brain, Cognition and Behaviour, Radboud University Medical Centre; Department of Research (N.K., V.W.), Sint Maartenskliniek; Departments of Sensorimotor Neuroscience (N.K.), and Neuropsychology and Rehabilitation Psychology (R.P.C.K.), Donders Institute for Brain, Cognition and Behaviour, Radboud University; Department of Medical Psychology and Radboudumc Alzheimer Center (R.P.C.K.), Donders Institute for Brain, Cognition and Behaviour, Radboud University Medical Centre, Nijmegen; Vincent van Gogh Institute for Psychiatry (R.P.C.K.), Venray; Klimmendaal Rehabilitation Center (R.P.C.K.), Arnhem; Tactus Addication Care (R.P.C.K.), Deventer; and Department of Rehabilitation (J.N.), Sint Maartenskliniek, Nijmegen, the Netherlands.

## Abstract

**Background and Objectives:**

Compensation strategies are essential in Parkinson disease (PD) gait rehabilitation. However, besides external cueing, these strategies have rarely been investigated systematically. We aimed to perform the following: (1) establish the patients' perspective on the efficacy and usability of 5 different compensation strategies; (2) quantify the efficacy of these strategies on spatiotemporal gait parameters; and (3) explore associations between the effects of specific strategies and patient characteristics.

**Methods:**

We recruited persons with PD and self-reported disabling gait impairments for this laboratory-based, within-subject study. Clinimetrics included the following: questionnaires (New Freezing of Gait Questionnaire, Vividness of Movement Imagery Questionnaire, Goldsmiths Musical Sophistication Index), cognitive assessments (Attentional Network Test and Montreal Cognitive Assessment [MoCA], Brixton), and physical examinations (Movement Disorders Society Unified Parkinson's Disease Rating Scale [MDS-UPDRS III], Mini-Balance Evaluation Systems Test, tandem gait, and rapid turns test). Gait assessment consisted of six 3-minute trials of continuous walking around a 6-m walkway. Trials comprised the following: (1) baseline gait; (2) external cueing; (3) internal cueing; (4) action observation; (5) motor imagery; and (6) adopting a new walking pattern. Spatiotemporal gait parameters were acquired using 3-dimensional motion capture analysis. Strategy efficacy was determined by the change in gait variability compared with baseline gait. Associated patient characteristics were explored using regression analyses.

**Results:**

A total of 101 participants (50 men; median [range] age: 66 [47–91] years) were included. The effects of the different strategies varied greatly among participants. While participants with higher baseline variability showed larger improvements using compensation strategies, participants without freezing of gait, with lower MDS-UPDRS III scores, higher balance capacity, and better performance in orienting attention also showed greater improvements in gait variability. Higher MoCA scores were associated with greater efficacy of external cueing.

**Discussion:**

Our findings support the use of compensation strategies in gait rehabilitation for PD but highlight the importance of a personalized approach. Even patients with high gait variability are able to improve through the application of compensation strategies, but certain levels of cognitive and functional reserve seem necessary to optimally benefit from them.

Gait impairment is common and disabling in individuals with Parkinson disease (PD). Reduced stride length, increased gait variability, and reduced arm swing are examples of continuous gait deficits that typically occur in persons with PD. As the disease progresses, episodic gait deficits, including freezing of gait (FOG) and festination, can also come into play.^1,2^ The presence of gait impairment often leads to falls and fall-related injuries and significantly affects functional mobility, independence, and quality of life.^3-5^

As dopaminergic medication and deep brain stimulation (DBS) usually have an only moderate effect on gait impairment, the application of compensation strategies has become an essential part of gait rehabilitation in PD.^6-8^ These strategies are typically self-invented by persons with PD and comprise a wide range of “detours” to overcome gait impairment and improve functional mobility. Examples include improved gait when walking to the beat of music, counting while walking, walking backward, climbing stairs, or when walking on a floor with a specific visual pattern.^9,10^ While often applied in the context of FOG, compensation strategies also improve continuous gait deficits.^11,12^

To date, compensation strategies in PD have usually been reported in the form of anecdotal case reports.^13-16^ With the exception of external cueing (e.g., rhythmic auditory stimulation), the efficacy of these strategies has rarely been investigated in a systematic manner. In 2019, a comprehensive framework of 7 distinct categories of strategies was proposed: external cueing, internal cueing, changing the balance requirements, altering the mental state, action observation or motor imagery, adopting a new walking pattern, and alternatives to walking.^9^ This framework served as the basis for a large-scale survey on the perception of compensation strategies in 4,324 persons with PD and gait impairment, providing Class IV evidence that compensation strategies are effective.^11^ However, the study also confirmed that the efficacy of specific strategies varies per person, highlighting the need for an individually tailored approach. It is still insufficiently understood what the underlying working mechanisms of these strategies are and which patient characteristics may be associated with the individual efficacy of the various compensation strategies. This is hampering the ability of healthcare professionals to provide much-needed personalized gait rehabilitation.

In this study, we evaluated the efficacy of 5 different categories of compensation strategies: external cueing, internal cueing, action observation, motor imagery, and adopting a new walking pattern. We had 3 aims: (1) to establish the patients' perspective on the efficacy and usability of the different strategies; (2) to quantify the efficacy of the strategies on spatiotemporal gait parameters; and (3) to explore whether the effects of specific strategies on gait are associated with certain patient characteristics.

## Methods

### Study Population

We predefined a target of 100 participants (grant proposal available on request). Participants were recruited from a large ongoing observational trial (PRIME-NL)^17^ and from ParkinsonNEXT (NL), an online recruitment platform for PD and parkinsonism research. Inclusion criteria were as follows: the presence of PD and self-reported gait impairment hindering usual daily activities. Exclusion criteria were as follows: comorbidity significantly affecting ambulation (e.g., stroke and orthopedic ailments); inability to walk unaided (or with a customary cane) for 3 minutes consecutively; severe auditory impairment hampering the perception of auditory cues; and severe cognitive impairment hampering the ability to comply to the study protocol.

Written informed consent was obtained from all study participants, in accordance with the principles of the Declaration of Helsinki. This study was approved by the local ethics committee CMO Arnhem-Nijmegen and the Institutional Review Board of the Radboud University Medical Center in Nijmegen, the Netherlands (Ref: 2019-5710).

### Experimental Protocol

In a 1-time study visit to the Radboud University Medical Center gait laboratory, participants completed 3 questionnaires, performed several clinical tests, and underwent a detailed gait assessment. Participants did not have to withdraw from their dopaminergic medication before the visit but refrained from taking renewed dosages of dopaminergic medication for the duration of the 4-hour visit. Consequently, clinical tests were performed in the dopaminergic ON-state, but gait assessment—at the end of the visit—was performed in “end-of-dose OFF.” We specifically opted for this approach because persons with PD typically experience most gait difficulties during this period, making it the clinically most relevant state to use any compensation strategy. Participants with DBS did not have to adjust their stimulation settings.

#### Questionnaires and Clinimetrics

Participants completed 3 questionnaires: the New Freezing of Gait Questionnaire,^18^ the Vividness of Movement Imagery Questionnaire,^19^ and an adapted version of the Goldsmiths Musical Sophistication Index (to quantify one's musical abilities).^20^ Cognitive assessment included the Montreal Cognitive Assessment (MoCA) as a measure of overall cognitive status,^21^ the short version of the Revised Attentional Network Test (ANT)—a computerized test measuring 3 attentional processes (alerting, orienting, and executive attention, expressed as network scores),^22^ and the Brixton Spatial Anticipation test as a measure of executive function—assessing rule detection and concept shifting (age-adjusted and education-adjusted percentile scores).^23^ These tasks have been used in PD populations before.^24^ A physical examination comprised the Movement Disorders Society Unified Parkinson's Disease Rating Scale (MDS-UPDRS) part III,^25^ the Mini-Balance Evaluation Systems Test (Mini-BEST),^26^ tandem gait (walking heel-to-toe in a straight line for 10 consecutive steps without taking balance correcting side steps),^27^ and the rapid turns test for FOG detection (making 3 360° turns in place, in both directions).^28^

#### Gait Assessment

Gait assessment consisted of six 3-minute trials of continuous walking around a 6-m instrumented walkway. The first trial always entailed the baseline gait condition, in which participants walked without applying any compensation strategies. The remaining 5 trials comprised the compensation strategy conditions in which patients applied the following: (1) external cueing; (2) internal cueing; (3) action observation; (4) motor imagery; and (5) adopting a new walking pattern. The remaining 3 categories proposed by Nonnekes et al.^9^ were not included: (1) altering the mental state (because it is difficult to control in a laboratory setting), (2) changing balance requirements (because it applies to turning and initiating gait), and (3) alternatives to walking (because gait variability is not an applicable outcome measure). The compensation strategy conditions were counterbalanced across participants, with the exception of motor imagery, which was always preceded by action observation. Participants were instructed to walk at a comfortable speed, and refrain from talking, consciously varying gait speed, or using a strategy other than the one specified.

The strategy choice within each category was based on feasibility: participants had to be able to apply them without extensive training, and they had to be easy to implement in daily life after the experiment. During external cueing, participants listened to a metronome (Metronome v1.2, Beijing Buluobang Co., Ltd., Beijing, China) and synchronized their steps to the beat. Metronome pace was customized by a trained researcher, matching or optimizing the participant's natural cadence as determined during baseline gait. Participants had the final say in determining the optimal pace. During internal cueing, participants silently counted in a rhythmic manner (e.g., 1-2-3-4-1-2-3-4) and synchronized their steps to the beat. During action observation, participants walked alongside a trained researcher and synchronized their steps. During motor imagery, participants consciously thought about the preceding action observation condition and visualized the researcher walking alongside them, synchronizing their steps. During adopting a new walking pattern, participants walked with exaggerated arm swing. Participants practiced each strategy until they felt comfortable.

After each trial, participants indicated whether the strategy had any subjective effect (positive, negative, or no effect compared with baseline gait). Finally, participants rated the probability of them continuing to use that strategy in daily life, using a 5-point Likert scale (1: very unlikely–5: very likely).

### Motion Data Acquisition and Analysis

Movement data were acquired using a motion capture system (VICON, Oxford, United Kingdom; sampling rate: 100 Hz). Sixteen markers were placed following the Plug-in Gait Lower Body Model.^29^

Strategy efficacy was determined by the difference in gait variability between baseline gait and each of the compensation strategy conditions. Gait variability was the predefined primary outcome because it is associated with fall risk in PD and other populations.^30-32^ Variability was expressed as the coefficient of variation (CoV) of stride time: stride time CoV = (SD stride time/mean stride time) × 100%. Stride time was defined as the time between subsequent heel strikes of the same foot and computed using a custom MATLAB script. Heel strikes were identified as (local) minima of the vertical displacement of the heel markers within the gait cycle. To negate the effects of the 180° turns (and associated deceleration/acceleration) at both ends of the walkway, only the center 3 m of the trajectory were included in the analysis.

### Statistical Analysis

Data analysis was performed in IBM SPSS 25 (SPSS, Inc., Chicago, IL). Group-level differences in gait parameters between baseline gait and gait with compensation strategies were examined using paired 2-tailed *t* tests with Bonferroni correction for multiple comparisons. For each strategy, the relationship between the predetermined primary outcome measure (change in stride time variability from baseline) and change in gait speed from baseline was assessed using Pearson correlation.

We investigated the association between participant characteristics and strategy efficacy using a 2-step approach. Exploratory analyses using unpaired 2-tailed *t* tests were conducted to compare the characteristics of responders (Q1 for improvement in gait variability compared with baseline gait) with those of nonresponders (Q4) for each compensation strategy, to identify potentially relevant variables. These variables were entered into a univariable linear regression analysis, adjusted for baseline gait variability. Finally, all associated variables were entered into a stepwise regression analysis with forward selection. The *p* value <0.05 was considered statistically significant.

### Data Availability

Data are available on reasonable request to the corresponding author.

## Results

### Study Population

We included 101 participants. Participant characteristics are outlined in Table 1, reflecting the desired clinical heterogeneity for the purpose of this study. Three participants did not complete all 6 gait conditions due to fatigue. Consequently, data on external cueing and motor imagery were available for 99/101 and action observation for 100/101 participants.

**Table 1:**
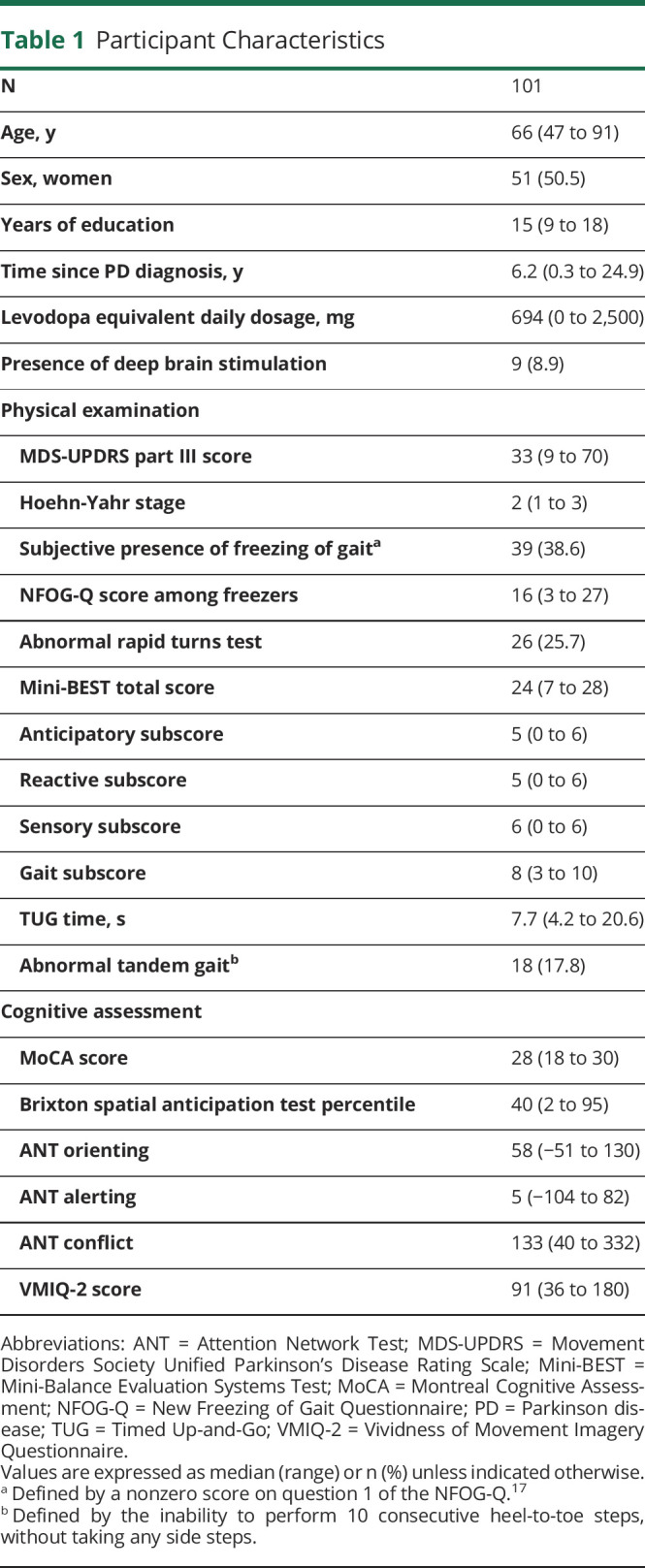
Participant Characteristics

Most of the participants (87%, 87/101) reported to have previously tried compensation strategies in daily life. The median number of strategies tried/currently used was 2, most often entailing internal cueing strategies (e.g., counting).

### Efficacy and Usability of Compensation Strategies

**Figure 1:**
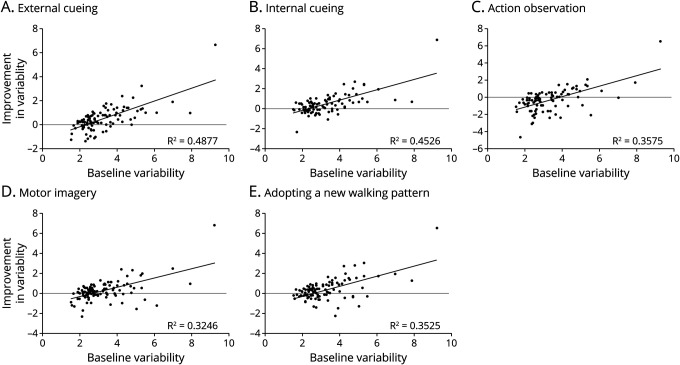
Association of Baseline Gait Variability and Improvement in Gait Variability With (A) External Cueing; (B) Internal Cueing; (C) Action Observation; (D) Motor Imagery; and (E) Adopting a New Walking Pattern The efficacy of the strategy is presented as the improvement in gait variability compared with baseline gait. Gait variability is defined as stride time variability, expressed by the coefficient of variation. Negative values correspond to an increase in variability compared with baseline, equaling a negative effect of the strategy.

**Figure 2:**
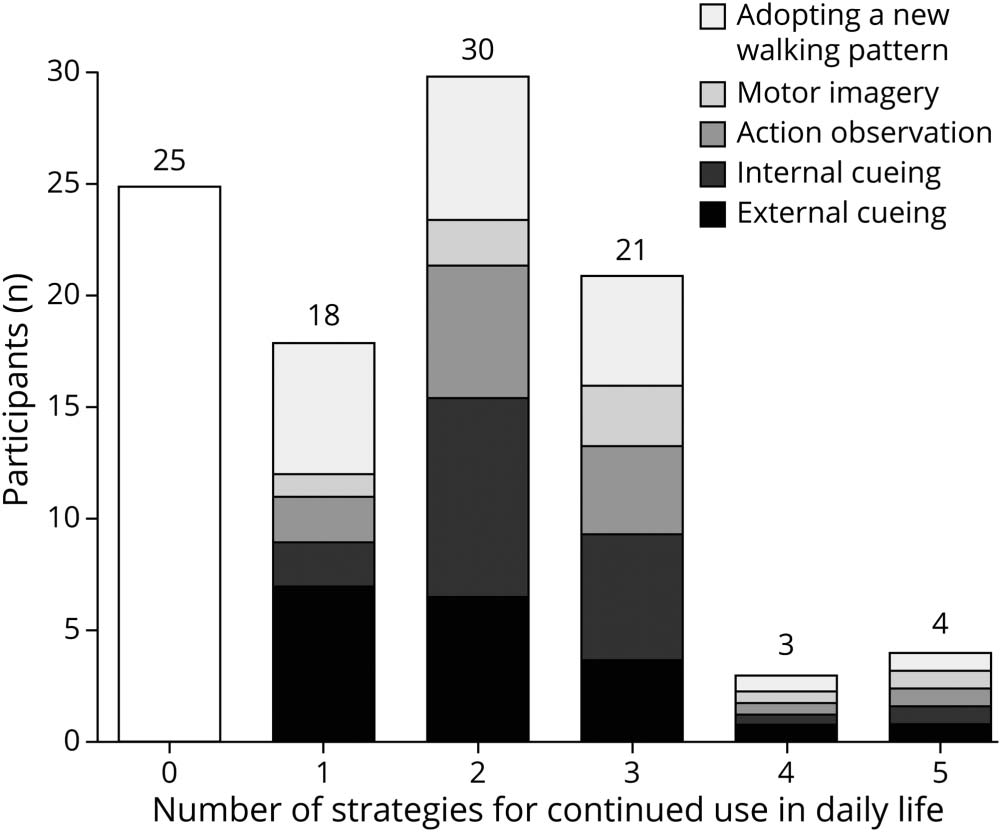
Participants (n) Who Were “Very Likely” to Continue Using a Number of the 5 Investigated Compensation Strategies in Daily Life Colors represent the strategies persons intended to continue using in daily life (e.g., in the group of participants who were “very likely” to continue using 1 strategy, this most often comprised external cueing or adopting a new walking pattern).

The efficacy and usability of the 5 compensation strategies are presented in Table 2. The effect of the strategies on spatiotemporal gait parameters varied greatly across participants (Figure 1), generating a relatively modest beneficial effect at group level. All strategies resulted in increased gait speed, predominantly due to an increase in stride length. While most strategies positively affected stride time variability (i.e., elicited a decrease in variability), action observation actually led to an increase in gait variability at group level. Overall patient-rated efficacy of the strategies was high, with the exception of action observation, which was most often considered to have no effect. Adopting a new walking pattern and internal cueing ranked highest regarding usability. Participants considered action observation to be the least usable strategy in daily life because it relies on the presence of another person. Figure 2 displays the number of participants who were “very likely” to continue using any of the investigated strategies in daily life. The median number of strategies for continued use was 2 per participant. Only 4% (4/101) would continue using all 5 strategies.

**Table 2:**
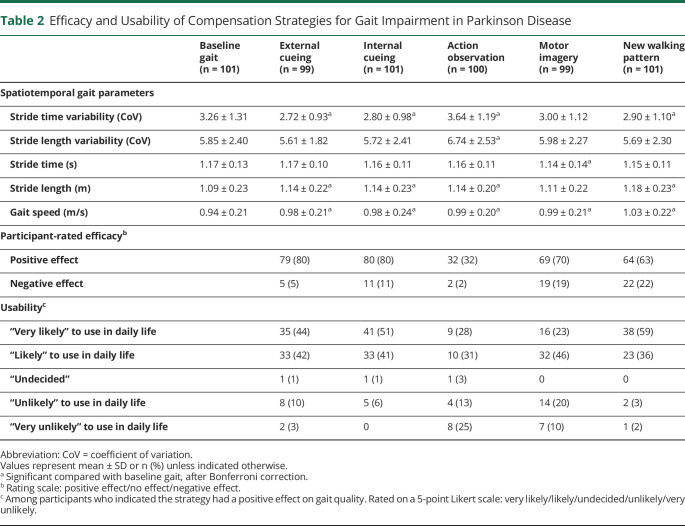
Efficacy and Usability of Compensation Strategies for Gait Impairment in Parkinson Disease

### Participant Characteristics Associated With the Efficacy of Compensation Strategies

For all strategies, the strongest predictor of efficacy was baseline gait variability (Figure 1). Participants with higher baseline variability (reflecting greater gait impairment) showed the largest improvements in gait variability while applying compensation strategies. For each strategy, the change in gait variability from baseline was linearly correlated with the change in gait speed from baseline (external cueing, internal cueing, adopting a new walking pattern: *p* < 0.01; action observation, motor imagery: *p* < 0.05).

Several other variables were associated with larger improvements, independent of baseline variability (Table 3). Participants with lower MDS-UPDRS part III scores (specifically PIGD items), higher balance capacity (higher Mini-BEST scores), faster Timed Up-and-Go (TUG) times, and better performance in orienting attention (higher ANT Orienting scores) showed greater improvements when applying strategies. Nonfreezers also showed larger improvements in gait variability compared with freezers. Strategy-specific associations with efficacy included higher MoCA score for external cueing and male sex for adopting a new walking pattern. All presented variables were entered in the stepwise regression analysis. Variables included in the final model are indicated in bold in Table 3. Coefficients of determination (*R*^2^) per strategy ranged between 0.419 for motor imagery and 0.647 for external cueing.

**Table 3:**
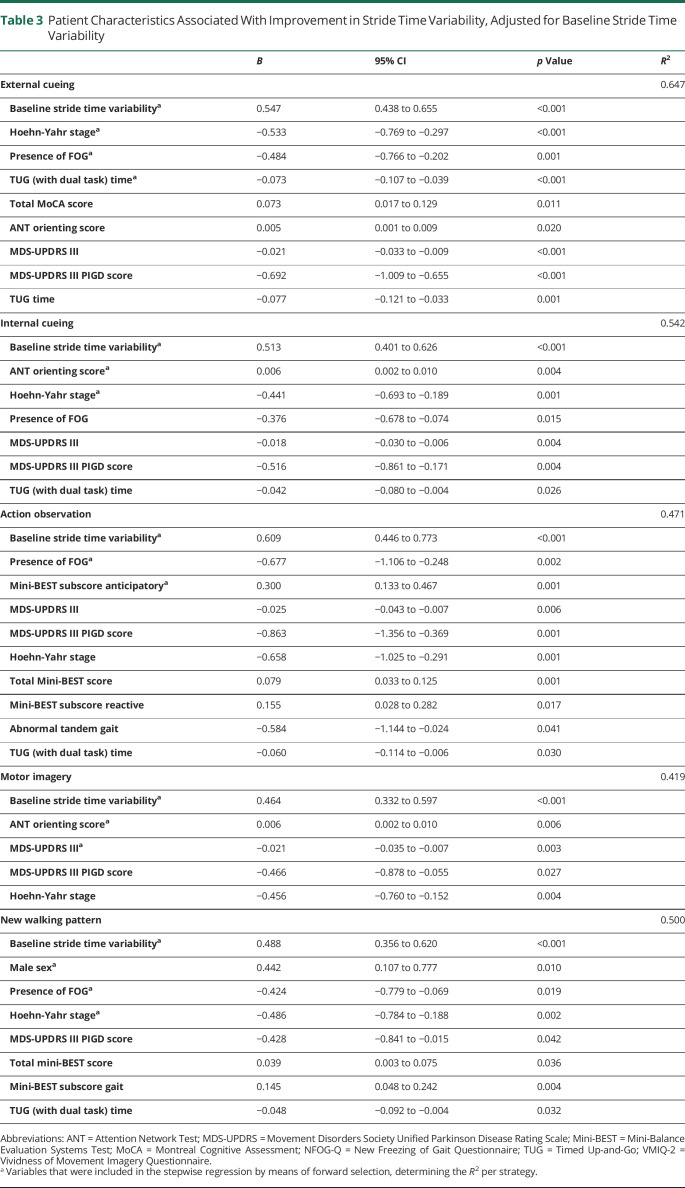
Patient Characteristics Associated With Improvement in Stride Time Variability, Adjusted for Baseline Stride Time Variability

## Discussion

We systematically evaluated the efficacy of 5 categories of compensation strategies (external cueing, internal cueing, action observation, motor imagery, and adopting a new walking pattern) in 101 persons with PD and gait impairment. Our main findings were as follows: (1) the beneficial effects on gait varied greatly across participants for the different types of strategies, highlighting the importance of an individually tailored approach to gait rehabilitation in PD; (2) a similar interindividual variation was noted for patient-rated efficacy and usability of the specific strategies, again highlighting a strong personalized element; (3) for all 5 strategies, higher baseline gait variability was associated with greater strategy efficacy, implying that persons with significant gait impairment are still able to improve gait quality by applying compensation strategies; and (4) the patient characteristics associated with the efficacy of specific strategies provide some insight into the possible underlying mechanisms of compensation and potentially explain why specific strategies seem to work better in certain patients.

Regarding the efficacy of specific compensation strategies to reduce gait variability, results varied greatly across individual participants. While 1 person showed dramatic improvement while using a certain strategy, the next would show no change or even an increase in gait variability when applying the same strategy. These individual differences are in line with the observations from clinical practice and are consistent with the results of a recently published survey study about the perception of compensation strategies in 4,324 persons with PD and gait impairment.^11^ Our findings emphasize the importance of trying out a variety of options to identify the optimal strategies for efficacy and usability for each individual patient. Using this approach in this study, 75/101 (75%) of participants was very likely to continue the use of at least 1 newly acquired strategy in daily life. However, only 4/101 (4%) of participants deemed all 5 strategies to be both effective and usable, again underlining the need to find an optimal personal fit. Trying out a variety of strategies is especially important, considering that patients will often require multiple strategies to perform their daily activities over many years. Even within 1 individual, the same strategy may have different effects depending on the situation or environment in which it is applied (e.g., indoors vs on a busy market square).^11^ In addition, although robust evidence is lacking, there are concerns that the efficacy of a strategy may taper off (or habituate) over time, necessitating a switch to alternative strategies.

Expectedly, the average baseline stride time variability of our participants was higher than the average reported for healthy adults of a similar age (mean ± SD: 3.26 ± 1.31 vs 2.20 ± 1.10).^33^ For all 5 strategies, higher baseline gait variability was associated with higher strategy efficacy. While it is certainly expected that persons with the largest baseline impairment have the greatest opportunity to portray the largest improvements, this finding contains an important clinical implication. Namely, persons with significant gait impairment are still able to improve gait quality by applying compensation strategies; that is, even among persons with the greatest gait difficulties, there is still room for improvement through compensation. This needs to be examined further in a population with more severely affected individuals. While participants all experienced hindering gait impairment, all were able to walk independently for at least 3 consecutive minutes, representing a group with relatively good functional mobility. Presumably, a certain level of functional and cognitive reserve is necessary to be able to successfully compensate for gait impairment.^34^ This is also supported by our finding that participants without FOG, with lower MDS-UPDRS part III scores, higher balance capacity, faster TUG times, and better performance in orienting attention demonstrated greater improvements in gait variability using compensation strategies.

The strategy-specific associations provide some insight into the possible mechanisms underlying compensation. It has been postulated that the application of compensation strategies ameliorates gait by facilitating a shift from automatic to goal-directed motor control, thereby bypassing the most affected basal ganglia circuitries.^9,35-37^ Moreover, their underlying mechanisms are hypothesized to at least partly differ for each category, potentially explaining why the efficacy of a specific strategy varies between patients.^9,11^ This is supported by a recent EEG study that presented distinct cortical correlates for external cueing, internal cueing, and action observation.^38^ We will highlight 3 interesting strategy-specific associations that we identified in this study.

First, participants with higher performance in orienting attention, that is, the ability to selectively attend to specific sensory input,^39^ showed larger improvements with external cueing, internal cueing, and motor imagery compared with participants with lower performance. This is in line with the presumed major role of attention in compensation for gait impairment, specifically in external and internal cueing.^9^

Second, a previous study on auditory cueing and the factors associated with increased gait speed in 39 nondemented PD patients revealed that persons with poorer cognitive flexibility, using the Wisconsin Card Sorting Test (WCST), showed the largest improvements.^40^ Using the Brixton Spatial Anticipation Test, similar to the WCST,^23,41^ we were unable to replicate this finding for improvement in gait variability. By contrast, we found better overall cognition (MoCA) was associated with larger improvements with external cueing. As proposed, this may be an indication that a certain level of cognitive reserve is imperative for successful compensation.^34,42^

Third, previous studies on auditory cueing in PD populations demonstrated an association between rhythmical ability and increased gait speed.^40,43^ Again, we were unable to replicate this association for gait variability. Years of musical training and self-perceived musicality (adapted Goldsmiths Index) showed no association with the efficacy of external auditory cueing in our population. Presumably, a more objective quantification of perceptual and motor timing abilities is necessary to reveal a potential connection to cueing efficacy.

In addition to the study limitations already discussed, several other points should be considered. First, the associated patient characteristics are specific to the strategy efficacy on gait variability and may have been different had a different parameter been selected. However, we specifically chose gait variability for its association with fall risk.^30-32^ Moreover, we found an evident correlation between the change in gait variability from baseline and the change in gait speed from baseline for each of the 5 strategies, which is important considering that patients often find gait speed one of the most important measures of their perceived gait quality.

Second, the associations are also specific to the strategy we selected to represent the category of compensation strategies as a whole (e.g., auditory cueing, rather than visual or tactile cueing in the category external cueing). Different strategies within a category of compensation strategies may have yielded different results. For example, while external auditory cueing seems to target temporal aspects of gait (e.g., stride time), external visual cueing more likely targets spatial aspects of gait (e.g., stride length) and may therefore appeal to a different type of patient.^44^ While the investigated strategies are a representation of the type of strategies that are usually evaluated by a physical therapist in clinical practice, persons with PD often use highly personalized strategies that may be a combination of strategies from different categories (e.g., counting while lifting the knees up high). In addition, imposed strategies may have a different (i.e., less outspoken) effect on gait compared with compensation strategies that are spontaneously invented by patients themselves.

Finally, the efficacy of compensation strategies is highly dependent on the context in which strategies are applied,^11^ so the reports of efficacy and the associated patient characteristics are specific to continuous gait in a laboratory-based setting. The laboratory-based setting may have particularly influenced the efficacy of action observation in this study. Because of the length of the walkway (6 m, with 180° turns on each end), participants were forced to walk alongside, rather than behind the person they were instructed to mimic. This meant they had to walk with their gaze directed to one side, rather than straight ahead. In addition, the need to take corrective steps to get back in sync after the 180° turns may have caused the detrimental effect on stride time variability at group-level. Presumably, continuous gait along a straight path may have led to an overall better response to the strategy at both the individual and the group level.

To conclude, these findings support the use of compensation strategies for gait impairment in PD, but underline the reality that one size does not fit all. The application of an individually tailored, personalized approach to gait rehabilitation is imperative to facilitate finding a suitable strategy for every person with PD.

## Glossary

ANT: Attentional Network Test
CoV: coefficient of variation
DBS: deep brain stimulation
FOG: freezing of gait
MoCA: Montreal Cognitive Assessment
MDS-UPDRS: Movement Disorders Society Unified Parkinson's Disease Rating Scale
Mini-BEST: Mini-Balance Evaluation Systems Test
PD: Parkinson disease
TUG: Timed Up-and-Go
WCST: Wisconsin Card Sorting Test
